# Public governance of medical artificial intelligence research in the UK: an integrated multi-scale model

**DOI:** 10.1186/s40900-022-00357-7

**Published:** 2022-05-21

**Authors:** Francis McKay, Bethany J. Williams, Graham Prestwich, Darren Treanor, Nina Hallowell

**Affiliations:** 1 Department of Population Health, The Ethox Centre and the Wellcome Centre for Ethics and Humanities, Nuffield, University of Oxford, Oxford, OX3 7LF England; 2 Department of Histopathology, St James University Hospital, Bexley Wing, Leeds, LS9 7TF England; 3 Yorkshire and Humber Academic Health Science Network, Unit 1, Calder Close, Calder Park, Wakefield, WF4 3BA England; 4 Department of Histopathology, St James University Hospital, Leeds, LS9 7TF England

**Keywords:** Public involvement, Medical research, Artificial intelligence, Governance, Citizen forums, Data access committees

## Abstract

There is a growing consensus among scholars, national governments, and intergovernmental organisations of the need to involve the public in decision-making around the use of artificial intelligence (AI) in society. Focusing on the UK, this paper asks how that can be achieved for medical AI research, that is, for research involving the training of AI on data from medical research databases. Public governance of medical AI research in the UK is generally achieved in three ways, namely, via lay representation on data access committees, through patient and public involvement groups, and by means of various deliberative democratic projects such as citizens’ juries, citizen panels, citizen assemblies, etc.—what we collectively call “citizen forums”. As we will show, each of these public involvement initiatives have complementary strengths and weaknesses for providing oversight of medical AI research. As they are currently utilized, however, they are unable to realize the full potential of their complementarity due to insufficient information transfer across them. In order to synergistically build on their contributions, we offer here a multi-scale model integrating all three. In doing so we provide a unified public governance model for medical AI research, one that, we argue, could improve the trustworthiness of big data and AI related medical research in the future.

## Plain English summary

How might the public be authentically involved in decisions about medical data sharing for artificial intelligence (AI) research? In this paper, we highlight three ways in which public views are used to improve such decisions, namely, through lay representation on data access committees, through patient and public involvement groups, and through a variety of public engagement events we call “citizen forums.” Though each approach has common strengths and weaknesses, we argue that they are unable to support each other due to a lack of proper integration. We therefore propose combining them so that they work in a more coordinated way. The combined model, we argue, could be useful for improving the trustworthiness of big data and AI related medical research in the future.

## Background

There is a growing consensus among scholars, national governments, and intergovernmental and non-governmental organisations of the need to involve the public in decision-making around the use of artificial intelligence (AI) in society [1–4]. Focusing on the UK, this paper asks how this aspiration for citizen inclusion in AI fits into the governance of medical AI research, that is, for research that involves training AI on data from medical research databases. The Health Research Authority defines a research database as “a structured collection of individual-level personal information, which is stored for potential research purposes beyond the life of a specific research project” [5]. Research databases are an increasingly important mechanism by which AI is trained on healthcare data in the UK, as evidenced by recent investments in medical image research databases [6–10].^1^

Public inclusion of medical AI research in the UK is generally achieved in three ways, namely, via lay representation on data access committees (DACs), through patient and public involvement (PPI) groups, and through deliberative democratic projects such as citizens’ juries, citizen panels, citizen assemblies, etc.—what we collectively call “citizen forums” [12, 13].^2^ Though historically each of these public involvement strategies have been enacted in various ways and to various ends, we discuss here some important criticisms in the literature to highlight apparent strengths and weaknesses of them and which stem from features commonly associated with each.

To make the case for our integrated model, we first outline the strengths and weaknesses. Given the extensive literature on public involvement, we do not aim to cover all the pros and cons of citizen forums, PPI groups, and DACs. Instead, we focus on what we see as three important issues relevant to the public governance of AI, namely, the extent to which public involvement is representative of the diversity of citizen views, whether there exists sufficient public understanding of medical AI research to make meaningful public dialogue possible, and whether opportunities exist for public contributors to impact the decision-making process. We summarise these issues under the headings of inclusion, informed deliberation, and influence respectively. Though generally important for public involvement [3, 14–16], we highlight these three as noteworthy for medical AI research for several reasons: first, because recent revelations of AI bias [17–19] demand inclusive approaches to AI governance; second, because AI is both complex and in flux, meaning that public understanding needs to remain contemporary in order to facilitate productive discussion; and third, because the ever-present possibility of involvement tokenism makes evidencing the impact of public views necessary (particularly in a field such as AI where public trust is often low).

Following our outlining of the pros and cons of citizen forums, PPI groups, and DACs, we develop our multi-scale model, showing how integration of these three domains of public involvement can provide an inclusive, informed, and influential public governance process for medical AI research generally.

## Main text

### Citizen forums

Since the 1970s, citizen forums—deliberative democratic events in which members of the public discuss issues of social importance to provide insight on public opinions, concerns, and interests—have become a popular method for engaging citizens on a number of social issues, for instance: electoral politics, state budgets, climate change, medical research, and new technologies including AI [4, 20–23]. Their primary contribution in these regards stems from their inclusivity. Developed within the context of a general participatory turn in democratic theory, citizen forums offer a way for lay publics to exercise their voice on issues that are not, strictly speaking, the purview of courts or expert stakeholders. In doing so, they provide opportunities for disagreements about collective goods to be democratically mediated, by providing summary consensus opinions on what is conducive to public interests [24–26].^3^

Practically speaking, however, not everyone can be involved in a citizen forum, and so the inclusivity at stake here is largely a statistical one [27]. Thus, forums generally aspire to collect the views of a *representative* lay public, selecting from a cross section of the citizenry through stratified sampling techniques in order to capture, as Ned Crosby, founder of the citizen jury method puts it, a “microcosm” of the community [28]. Stratified sampling here may be done according to traditional demographic criteria such as gender, age, ethnicity, etc., or via other metrics that enable a broad representation of views on a topic—what one might call viewpoint representation rather than demographic representation.

Citizen forums are a well-regarded means by which public views can be included in decision-making on issues of broad social importance, and they have been used to that end in order to think through AI’s role in society [4]. In that sense, they can be said to be strongly inclusive. They are potentially weaker, however, in terms of informed deliberation and influence. As the political theorist Albert Dzur [19] describes, citizen forums exist in a kind of “civic limbo” (p. 43). Neither citizen owned, nor citizen led, they are instead usually designed by academic researchers and thinktanks as ad hoc experiments in deliberative democracy. This fact may have multiple consequences, including abstract forms of commitment (participants are paid to have opinions on topics they might otherwise be indifferent to), lack of public accountability (participants are randomly selected, rather than elected), and constraints on discourse (researchers, not participants, set the terms of debate). More importantly, it also impacts how informed its members are about the topic under discussion and how much influence they may have to change outcomes.

Regarding the former, forum participants often undergo preparedness training on their selected topic in order to facilitate productive discussions, for instance, through being given informational materials before the event or by being provided with opportunities to listen to and speak with expert stakeholders about the topic. In being formed ad hoc, however, forums tend to be utilised on a one-time basis. Lacking existence beyond their one-time use, members generally have few opportunities to develop a political identity around the question at hand, and therefore to learn and evolve beyond their participation in the forum itself. Indeed, forum members may spend up to a week or so reflecting on their chosen topic, at which point they offer their summary consensus views and disband.

The relative lack of opportunities for political identity formation, and the limited chances for learning and memory in regard to the topic at hand is a problem for public involvement strategies that require maturation of their views over time. It is especially important, however, for the governance of new technologies (like AI) where the rapid pace of development, and the broad yet unpredictable ways it can impact society, means ongoing monitoring of research and the outcomes of data sharing decisions is important [29].

The problem justifies the need for what Understanding Patient Data (UPD) have called a “learning governance” approach to data-driven technologies [30]. Inspired by the concept of the “learning healthcare system” [31], in which iterative feedback loops exist between the outcomes of healthcare decision-making and the decision-making process itself, the idea here is to monitor the outcomes of decisions regarding data sharing to learn from what has worked and what has not. UPD posit citizen forums as a point in that learning governance system: monitoring outcomes and reflecting on them in relation to public values. Building on Dzur’s point, however, we may say that, as they stand, citizen forums are often not well designed for that task since they commonly lack opportunities for ongoing learning and thus for the organisational memory that sustains the monitoring processes.

Regarding influence, citizen forums are also not well-integrated into existing medical AI governance mechanisms, which is more commonly the purview of PPI groups and DACs. Since they require specialist knowledge and extensive resources to hold, they are not routinely employed as a governance strategy by medical researchers. Indeed, as one-time ad hoc events, it is unclear whether they can be properly said to be part of a public governance function in the first place. When they are held, they play a role largely through the literature produced as outputs of a forum. Such reports or articles may or may not influence institutional decision-makers, depending on whether they reach a wide audience and persons with decision-making capacity willing to heed their recommendations. Consequently, forums can be said to have weak influence in terms of public governance of medical AI research, since being external to, or on the periphery of, formal governance processes, they lack official mechanisms by which their advice can impact those who make decisions regarding the sharing of medical data. As Street et al. concluded in their review of citizen juries for health policy decision making, for instance, “few juries' rulings were considered by decision-making bodies thereby limiting transfer into policy and practice” [27].

### PPI groups

PPI groups—also known as patient and public advisory groups, patient reference groups, patient participation groups, etc. [32]—are a further mechanism by which patient experiences and public values are brought into medical AI research governance. The functions of PPI groups overlap, to some degree, with citizen forums insofar as both are deliberative spaces used to advise medical experts on decision-making. They differ from citizen forums, however, in a couple of ways.

First, they tend to focus on “typical” rather than “statistical” representation. As Fredriksson and Tritter [33] note, statistical representation refers to the goal of obtaining a representative sample of the total population (the usual aim of citizen forums). By contrast, typical representation means recruiting participants based on the possession of a shared experience or characteristic. At its broadest level typical representation, in the case of PPI groups allied with health research projects, means involving *patients*, that is, persons with experience as health service users, a wide category that includes most members of the public. Construed more narrowly, however, it may mean having experienced a specific illness or condition, such as being a cancer survivor in the case of cancer research. Alternatively, PPI members may be recruited by virtue of their status as a public involvement expert—since recruitment often happens through informal PPI networks, membership in one PPI group can facilitate membership in another—or by being representative of other direct and indirect stakeholders: carers, independent patient groups, charitable organisations, local community groups, etc. PPI as a collective term covers a range of potential subject positions, therefore. As such, they can elucidate a wide array of collective interests, whether it be those of patients as a general category, those of specific patient communities, various community groups classed according to demographic criteria (e.g., by ethnicity, age, gender, etc.), or the general interests of concerned citizens. There are some challenges, however, that PPI groups may face. For instance, where PPI groups focus on patient experience, they need to be careful to avoid conflating patient and public values. This is important because, as Fredriksson and Tritter further note [33], patients and publics have functionally different roles, the latter providing collective perspectives on societal (i.e., public) interests (the kind deliberated upon by citizen forums), the former providing anecdotal perspectives on sectional (i.e., vested) interests. Having patient experience serve as a proxy for public values risks ignoring this differentiation and limits the potential for the public governance of medical AI research in the process.

Even where this is not the case, however, PPI groups are also often criticised for lacking demographic diversity, disproportionately favouring, as multiple studies argue, white, older, middle class, and well-educated individuals [34–36]. Though in theory PPI groups may provide insights across a range of collective interests, and may make earnest attempts to embrace multiple viewpoints and positions, they can often fail in their aspiration for demographic diversity and thus for capturing broad societal interests.

Despite these shortcomings, PPI groups could nonetheless be said to be moderately inclusive in comparison to citizen forums. Though they do not recruit for statistical diversity, and sometimes risk conflating patient and public interests, they do provide opportunities for vested stakeholders to be involved in medical governance processes, and they are a well-established mechanism for that. Indeed, in England the integration of PPI into medical governance has been central since the establishment of Community Health Councils in 1974, following which has been a series of successive laws that have evolved the landscape of PPI in healthcare, first through the development of Patients forums and the Commission for Patient and Public Involvement in Health in 2003, and then by local involvement networks in 2007 [37–39]. Since 2012, the Health and Social Care Act (2012) has given NHS England and Clinical Commissioning Groups a legal duty to involve the public in service planning, proposals, and decisions, and set up local Healthwatch to involve the public in that process by understanding their needs and experiences [34, 37]. Though not having a strictly legal duty, public involvement has also extended beyond commissioning of services to become central to health research, with the Health Research Authority—the body that regulates medical research—and the National Institute for Health Research—the body that funds applied medical research—both advocating a central role for PPI as part of the policies [34, 40]. In that sense they mirror multiple similar endorsements for PPI in health care and research, around the world [41–43].

PPI groups are also stronger in terms of informed deliberation in comparison to forums, due to possessing relatively stable lay membership structures. That is to say, since participants are able to contribute to the group over the long term, they can observe research projects throughout their life span. In that sense they are able to develop the organisational memory that sustains a learning governance approach. Moreover, because of the long-standing presence of their members, and their integration into medical research governance practices, PPI groups have a stronger potential for influence. Though PPI groups (like citizen forums) are generally *advisory* bodies, evidence suggests they can contribute to research design and outcomes where they are well conducted and supported, and where there are authentic opportunities for members to be involved [44–47]. Moreover, since members often build up stable ties with organisational decision makers, this can give rise to informal obligations for decision-makers to feedback their outcomes to PPI groups, providing accountability from medical experts to patient views through obligations to listen and respond.

### DACs

Finally, DACs provide an alternative means for public governance of medical AI research, though to what degree is unclear. This is because DAC structures and functions are much less elaborated upon in the literature in comparison to either citizen forums or PPI groups. Partly this is due to their relative novelty as a governance mechanism (many DACs are building capacity and capability in public governance) and partly it is due to the fact that, as Shabani et al. note, there is little transparency around DACs overall [48], making it hard to determine membership processes. Some mainstream examples, for example, the Managing Ethico-social, Technical and Administrative issues in Data ACcess (METADAC) and the Mahidol Oxford Tropical Medicine Research Unit (MORU), include public members on their committees as part of their oversight procedures [49], others, such as Optimam [50], have lay representation on a closely related body such as a steering committee, while others do not have them at all and may rely instead on information governance experts to represent public interests. We agree with a recent report by Health Data Research UK [51] that DACs need public facing members on their committees. Insofar as they do, they provide an important means for public governance of medical AI research.

How then do DACs fair in terms of inclusion, informed deliberation, and influence? Regarding inclusion, DACs can be said to be weakly representative as, even when they do include lay members, they generally do not recruit for statistical diversity. Practically speaking, DACs tend to be limited in the number of lay participants they can accommodate. Sandler and Basl [52] suggest a hypothetical committee with two citizen participants, a number that is confirmed by existing examples [53]. A survey of UK Health Data Research Alliance members notes more than two lay members in some cases, with one larger committee containing nine [54], though in general they recommend a minimum of two for a well-functioning DAC [51]. However, they have stronger potential for informed deliberation in comparison to citizen forums (and possibly PPI groups depending on how they recruit for typical representation). This is because DACs, like PPI groups but unlike forums, have opportunities for ongoing membership and thus for the learning and memory that comes with that. Not only that, but given the complex rules around sharing data, DACs may be more likely to seek out public representatives with enhanced expertise regarding data governance issues to facilitate nuanced discussion of applied data sharing issues. Lay DAC members may thus possess a dual identity as both a data expert and a patient representative, further establishing their strength in terms of informed deliberation. Both forums and PPI groups may also recruit members with such dual expertise, but insofar as they commonly aim to acquire a range of viewpoints, whether considered statistically or typically, it is less likely a feature needed for them. By contrast, DACs may face internal pressures to find lay DAC members that are comfortable with the technicalities of AI in order to facilitate in depth discussion of data sharing issues.

Finally, DACs are arguably stronger in terms of influence in comparison to both forums and PPI groups, insofar as they are gatekeepers for medical AI research (researchers wanting access to data go through them by default). Evidence that exists on DACs suggests that all members, including lay participants, share responsibility for the decision-making regarding whether and how researchers get access to medical data [54]. This is not usually the case for forums and PPI groups, however, both of which tend to be advisory.

### A multi-scale model of AI governance

Table 1 summarizes the above relative strengths and weaknesses of citizen forums, PPI groups, and DACs in terms of their possibilities for inclusion, informed deliberation, and influence. The table highlights tendencies, rather than universals, though they are tendencies general enough to be worth noting.

**Table 1:** Relative strengths and weaknesses of public involvement initiatives

	Citizen forums	PPI	DACs
Inclusion	Strongly inclusive insofar as they aim for statistical representation through stratified sampling techniques	Moderately inclusive insofar as they aim to capture views of a range of vested stakeholders, but risk conflating patient and public interests and often fail to achieve demographic diversity	Weakly inclusive insofar as they generally accommodate one or two lay members
Informed deliberation	Weak where, due to their ad hoc design, they lack the potential for learning governance	Strong due to stable membership structures which support learning governance	Strong due to stable membership structures and because the dual expertise of lay members may support informed decision-making
Influence	Weak as they lack formal integration into medical research governance	Strong because of documented impact on research design and outcomes and because members build up ties with expert decision-makers, who, though obligations of reciprocity, are held informally accountable to the PPI group	Strong as lay members have equal decision-making powers regarding data sharing for medical AI research

What is interesting about the above is the relative complementarity of those strengths and weaknesses: citizen forums are strong in terms of inclusion insofar as they provide deliberated insights from statistically representative groups of lay publics, but they are weak regarding informed deliberation and influence in instances where they have limited learning opportunities and no formal links to existing medical governance mechanisms. PPI groups, by contrast, are less inclusive insofar as they capture vested stakeholder interests but often fail in terms of demographic diversity. They are stronger, however, for informed deliberation due to possessing relatively stable membership structures, which allows for learning and memory. They are also stronger in terms of influence due to their documented impact on research design and outcomes and because of the informal bonds of reciprocity that build up between public members and experts, making the latter accountability to the former through feedback. Finally, DACs are weakly inclusive due to the smaller numbers of lay representatives they practically accommodate. They are stronger, however, in terms of both informed deliberation and influence due to the possibility of retaining members over the long term (including members with expertise in data sharing) and due to the equal decision-making powers of their members.

**Fig. 1:**
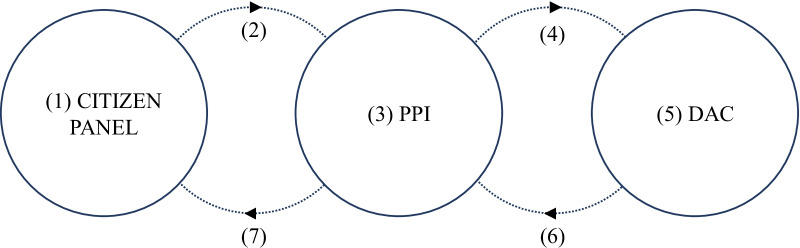
A Model of Multi-scale Governance. The process begins with citizen forum members meeting to deliberate uses of medical data by AI researchers and to monitor outcomes of prior data sharing to make sure it aligns with public values (**1**). Citizen forum members then feedback their consensus opinions to the PPI group (**2**). PPI group members review those recommendations (**3**) and advocate for them to the DAC (**4**). Lay DAC members in turn advocate for forum recommendations received via the PPI group to bring devolved power to citizen deliberations (**5**). Completing the loop in the other direction, DACs may instigate forum deliberations by suggesting topics requiring debate and citizen input (**6**). The PPI group contribute to this process by co-designing and planning future forums based upon the broad remit provided by the DAC (**7**)

Due to the complementarity of strengths and weaknesses, forums, PPI groups, and DACs could likely provide a well-rounded approach to public governance of medical AI research if they could work together. For the most part, however, they lack that integration (members are not institutionally organized to easily speak to and learn from each other). Though integration may occur via informal mechanisms at times, insofar as citizen forum, PPI and DAC members may move across involvement activities and communicate their learning in the process, relying on such informal mechanisms is not a substitute for a transparent method enabling the translation of citizen views into organisational decision making. In order to synergistically build on their contributions, then, we suggest integrating them in a multi-scale model of medical AI governance. Figure 1 provides an overview of that model, beginning with the citizen forum.

### Unpacking the model

Since our model represents an iterative process, there is no formal starting point. For sake of explanation, however, we begin with the problem of inclusion and thus the role of citizen forums. If medical AI governance is to take seriously the goal of public inclusion, there needs to be some way of capturing broad, democratically and deliberatively mediated public interests regarding medical AI data sharing. Since citizen forums are strong in terms of public inclusion, they could maintain this primary function by deliberating upon public interests regarding uses of medical data by AI researchers and by monitoring outcomes of prior data sharing to make sure it aligns with public values. In doing so, they can fill in the gap left over by PPI groups and DACs, both of which are relatively weaker in terms of inclusive debate.

Since, however, forums have weak potential for learning, recruitment for them need not just be for representative diversity, but also for longevity. This means extending the life of the citizen forum beyond its usual one-time use and committing to its ongoing existence as part of an oversight framework for medical AI research. This could be achieved either by research projects costing for their own ad hoc standing forum, or it could be achieved centrally in the form of a national medical AI citizen panel. We discuss the benefits of the latter at the end (see section “Questions”). In either case, however, the establishment of such a forum would enable a representative public, with relatively stable and ongoing membership, to promote political identity, learning, and memory around the topic of medical AI in order to strengthen the potential for informed deliberation.^4^ Such a group would meet periodically (the exact frequency would depend on need and resources) to deliberate issues regarding medical data sharing and to review outcomes of previous data governance decisions for medical AI research so as to provide ongoing monitoring.

As mentioned, however, forums lack influence as they are not integrated into existing medical research governance. To strengthen the potential for their deliberated insights to influence decision makers, therefore, such a forum would need to be supported through PPI and DAC activities. This would mean enhancing the functions of PPI groups so that they liaise with citizen forum members and advocate for their summary recommendations. Here we should note that it is expected that forum recommendations may be of such a generality that it is unclear how they might be applied to medical research contexts. PPI members could play a further important role in this regard, therefore, helping to facilitate the translation of citizen forum recommendations into practical medical settings, so as to make concrete governance recommendations that are attuned to the exigencies of a complex organisation like the NHS. From there the PPI group can present their applied advice to the DAC whose lay members are duty bound to advocate for those recommendations to bring a devolved perspective to citizen views.

All this represents just one direction in which the model works. It is important to note, however, that the influence flows in the reverse direction as well, that is, from DACs to the forum. Since DAC members are close to data access and medical AI research requests, they are well placed to instigate the deliberative process around issues they feel unsure to decide upon. Hence, they can initiate citizen forum meetings by asking the PPI group to plan and execute a forum based on issues that they believe require broader public involvement. This gives the PPI group a further opportunity to help co-design the forum based on the broad remit set by the DAC.

### Benefits

The above model has a number of benefits over the unintegrated approach to public governance of medical AI as it currently stands. First, is its inclusivity. As mentioned at the beginning of this paper, one challenge for medical AI is how to include broad citizen views into governance decisions. By integrating citizen forums with PPI groups and DACs, the model offers a way of overcoming the longstanding challenge of a lack of public diversity in the public governance of medical research.

Second, it brings a learning governance perspective to that process by recruiting forum members over the long term. This is important because, as is commonly recognised, AI is new, meaning that we do not yet have a full picture from data to outcomes on what AI can do. Hence, governance approaches are needed that allow for learning and monitoring of the outcomes of previous data sharing decisions. It is also not without importance that public views may shift over time, and our learning model supports that possibility.

Third, the model strengthens citizen influence over medical AI research decisions. As mentioned, forums lack mechanisms by which their recommendation may impact decision-makers. Our model provides a pathway for that by distilling citizen forum recommendations through PPI groups, so that they might be taken up by DAC members. This devolved approach to public governance is preferable to relying simply on PPI members and DACs, since it removes the burden from them for determining public interests based on their sectional interests. In that sense, it brings a wider and potentially more representative approach to public governance of medical AI.

### Questions

The idea of a multi-scale model raises multiple questions regarding its feasibility and how it might work in practice. It is not possible to address all those questions beforehand since there are different ways the model may be enacted. As mentioned, for instance, forums might be integrated via ad hoc standing panels set up by individual research projects or could be addressed centrally through a national medical AI citizen panel. We address here, however, some important questions to consider and highlight the direction we think the model ought to take going forward.

#### Is a multi-scale approach necessary for every medical AI project?

The model is an invitation and not a requirement. It is offered as a way for medical AI researchers to strengthen their public governance strategies regarding inclusion, informed deliberation, and influence. In being abstract, it also allows for different ways of implementing it. Hence, it would be up to research projects to reflect on their need for adopting it and to adapt it to those needs.

#### How feasible is this model given that it may require large infrastructural changes and high costs?

The model builds upon systems that already exist in various ways. For instance, DACs and PPI groups are already well-established public governance procedures, and changes to them come largely from augmenting the duties and responsibilities their members possess, by providing such members with, for instance, informal powers to co-design and review forum deliberations. This may require additional time on behalf of those members, which would need to be costed for as part of ongoing public involvement activities.^5^ The biggest change and burden would come from the integration of citizen forums. Citizen forums are costly and integration with them may be outside the limited resources of project-based funding. Low resourced medical AI research projects may therefore lack capacity or capability to design and hold a forum, though well-resourced research projects may find it possible and desirable. In the former case, a national medical AI citizen panel could provide an overarching solution, as once established it could be run as a service at cost to organisations, reducing the financial and time burdens associated with setting up a forum.

#### What status would forum decisions have? What happens when disagreements occur across the levels and who is the final authority?

We see forums as providing consensus recommendations based on what Kieran O’Doherty has called “deliberative public opinion” [26]. Given their ontological status as deliberated opinion, their consensus recommendations should be taken as providing insights into public preferences and interests. They are to be listened to carefully, though PPI groups may find it important to qualify or disagree with them if they have good reasons to do so. If so, there should be an expectation for a transparent response with reasons for the divergence. Indeed, we would expect transparency across the multi-scale model, through publication of group members, deliberations, minutes, decisions, etc. This would be especially important if implementing a centralized national forum, though it would be prudent for ad hoc standing panels as well. As DACs are gatekeepers for sharing researcher, they would have final authority, again, with the expectation of transparent response when disagreements are had.

#### How could a national forum be established? Who would lead and own it?

The idea of a national panel is one way in which the multi-scale model could be enacted. It could also be enabled, as mentioned, in a more fragmentary form, by research teams costing to set up their own ad hoc standing forums. A national forum, however, would provide a way to centralise that work, cutting down on the financial and time burdens research projects need to commit when soliciting public views. This would be especially beneficial for low-resourced research teams, though we suspect it would be of benefit to others due to efficiency and consistency regarding public recommendations. There are, however, multiple challenges with establishing such a forum. For instance, how could it be established? Who leads it and runs it? Is it as inclusive as it sounds or could it be seen as a way to exclude members of the public by other means, for instance, by imposing a centralised, top-down model?

The idea of the national panel is offered as a provocation. To answer these questions, we suggest the most appropriate next step would be multistakeholder dialogue assessing its desirability and feasibility. There is a large network of stakeholder organisation relevant to discussions of health research, medical data sharing, and public involvement—e.g., NHSx and NHS digital (now part of Transformation Directorate at NHS England), HDR UK; NIHR; Use MY data; DATA-CAN; the James Lind Alliance; the National Data Guardians Office, etc. We recommend therefore that an organisation like NHSx lead a multi-stakeholder consultation on the possibility of such a panel to develop the approach and to answer those questions.

#### How do the exigencies of the covid-19 pandemic affect the development/application of the model in practice?

Over the past couple of years, responses to the covid-19 pandemic has affected possibilities for public involvement in a number of ways, from reducing the likelihood of researchers seeking public involvement to transforming the means through which deliberation occur [55–57]. Some of these changes—for instance, the transition to digital methods of involvement—can be enabling for some, but constraining for others, depending upon facility with and access to digital communications. Nonetheless, the possibility of digitalised participation could provide an opportunity for both ad hoc standing forums and national panels by providing an online gateway connecting participants over geographical distances and beyond the confines of in-person meetings. It can also limit some of the costs with in-person events. Of course, organisers would need to be mindful of the possibilities of new forms of digital exclusion arising as a result of such moves and find ways to maintain the commitment to inclusion that is central to public involvement.

## Conclusion

It has been argued that one reason why medical data sharing projects sometimes fail to obtain public support stems from insufficient public involvement [58]. Despite growing calls to involve the public in decision-making around the use of artificial intelligence, however, without sufficient integration across the multiple modalities of public involvement, medical AI research is likely to fail either in terms of inclusion, informed deliberation, or influence. We argue that our multi-scale model, grounded in the views of a national medical AI citizen panel as mediated by PPI groups and DACs, provides a way of addressing that challenge and thus of improving the trustworthiness of medical AI research in the future.

## Data Availability

Not applicable.

## Abbreviations

DACs: Data access committees
PPI: Patient and public involvement

## Footnotes

1 Other examples of research databases can be found at the Health Data Research Innovation Gateway [11].

2 One could also add here lay representation on research ethics committees (RECs). RECs, however, exempt research databases from review where “generic” ethical approval already exists for the database and when the data being shared is de-identified. Due to the availability of REC exemptions, we focus on citizen forums, PPI groups, and DACs for this paper, reserving the question of whether and how RECs might be integrated into the multi-scale model for a future analysis.

3 Though the idea of public interest is controversial (there are multiple publics with multiple interests), forums are nonetheless one of the few means currently available by which collective goods can be deliberated about in the public sphere to inform decision-making. That there is diversity in public opinion is not necessarily problematic, as participants are asked to deliberate not in terms of their personal preferences, but on behalf of the common good (RSA 2018).

4 This does not mean that membership cannot change over time. As with any organisation, people may leave, others may join. Practically speaking, it may be advisable to have a pool of participants to draw on, with the intention of recruiting others when members leave in order to maintain representativeness.

5 The question of whether participants should be paid for public involvement work is a debatable one. We see it as ethical and practical, however, to return value to members for their contributions, though whether this value is returned in a financial form is an open question. As a default position, we think some form of financial renumeration is important to cover costs and to incentivise people to participate and suggest following NIHR guidelines on payment for PPI.
